# The Interactive Model of L2 Listening Processing in Chinese Bilinguals: A Multiple Mediation Analysis

**DOI:** 10.3389/fpsyg.2022.871349

**Published:** 2022-04-08

**Authors:** Yilong Yang, Guoying Yang, Yadan Li

**Affiliations:** ^1^Research Center for Linguistics and Applied Linguistics, Xi’an International Studies University, Xi’an, China; ^2^School of English Studies, Xi’an International Studies University, Xi’an, China; ^3^Ministry of Education (MOE) Key Laboratory of Modern Teaching Technology, Shaanxi Normal University, Xi’an, China; ^4^Shaanxi Normal University Branch, Collaborative Innovation Center of Assessment Toward Basic Education Quality at Beijing Normal University, Xi’an, China

**Keywords:** L2 listening, spoken word segmentation, cognitive inhibition, cognitive flexibility, the interactive model

## Abstract

Second language (L2) listening is a common challenge for language learners. It remains largely unknown how bilinguals process L2 listening. The literature has suggested an interactive model of L2 listening processing. However, few studies have examined the model from an experimental approach. The current study tried to provide empirical evidence for the interactive model of L2 listening processing in bilinguals by exploring the relationships among English spoken word segmentation (SWS), cognitive inhibition, cognitive flexibility, and L2 listening proficiency. The results showed positive associations among SWS, cognitive inhibition, cognitive flexibility, and L2 listening proficiency. Mediation analysis suggested that SWS might have a positive influence on L2 listening proficiency both directly and indirectly through cognitive inhibition and cognitive flexibility, respectively. These results imply that both bottom-up (reflected at SWS) and top-down (reflected at cognitive inhibition and flexibility) processes are engaged in bilinguals’ L2 listening processing.

## Introduction

Listening is an indispensable prerequisite for us to sustain effective communication. It constitutes forty-five percent of our total communication (Feyten, 1991). Unlike reading, it remains an obstacle for language learners to identify and segment L2 utterances into understandable segments during oral communication because there are no obvious signs, pauses, or punctuations signaled within a complete and fluent speech flow (Cole and Jakimik, 1980). Additionally, listening to a language that is rhythmically different from one’s first language (L1) can be particularly challenging (Vandergrift, 2008), for it requires second language (L2) listeners’ to carry out additional processes to overcome comprehension barriers (Flowerdew and Miller, 2005). Moreover, listening provides the basis for the development of the other main language skills, i.e., speaking, reading, and writing (Murphy, 1991; Vandergrift, 1997; Fotos, 2001; Snow, 2005; Hinkel, 2006). L2 listening, therefore, has been considered lying at “the heart of second language learning” (Vandergrift, 2007).

### The Interactive Model of Second Language Listening

Given the importance of L2 listening, scholars have made continuous efforts to unravel the underlying mechanisms of L2 listening processing. Several models have been developed from both the linguistic and cognitive perspectives, namely, the bottom-up and top-down models of L2 listening processing.

The bottom-up model of L2 listening processing was first developed in the 1940s and 1950s under the influence of behaviorism. It follows the traditional idea that communication is a means of information transmission; listeners accrete each basic linguistic unit (e.g., individual sounds or phonemes) within the speech into increasingly larger meaningful units, e.g., clauses, sentences, or discourses (Vandergrift, 2011). The bottom-up listening processing involves decoding. It indicates that the listeners have to segment the speech into meaningful units during communication (Vandergrift, 2011). Studies have revealed that L2 learners are more prone to segment speech by invoking their L1 segmentation procedures (Cutler, 2000), and this phenomenon is more prominent in low L2 proficiency listeners (Goh, 2000; Graham, 2006). Evidence has also shown that listeners with lower L2 proficiency need to put more effort into the bottom-up processing than those with higher proficiency (Li et al., 2020; Yang et al., 2021). Field (2008) even ascribed the failure of L2 listening comprehension to the incorrect segmentation of speech by L2 listeners. These studies together imply that spoken word segmentation (hereafter SWS) is an important way of bottom-up processing in successful L2 listening. Although it is plausible that the pure bottom-up model explains the mechanism of language learners listening and combining discrete segments to form meaning, it cannot account for the situation that listeners may still achieve successful listening comprehension without having to identify every single word of the interlocutor’s utterance.

Another well-established model is the top-down L2 listening model. It appeared in the 1980s under the influence of constructivism, following the concept that considers listening as a purpose-driven process (Flowerdew and Miller, 2005). The top-down listening processing includes three main categories of listening strategies, i.e., metacognitive strategies, cognitive strategies, and socio-affective strategies (Vandergrift, 2008). Previous studies have stressed the importance of metacognitive strategies as the chief listening strategies used by language learners to successfully comprehend L2 speech (Namaziandost et al., 2019). Another study has shown a significantly heavier use of metacognitive strategies by L2 listeners with higher proficiency than novice L2 listeners (Vandergrift, 1997). Therefore, metacognitive strategies seem to play a pivotal role in L2 listening. Since metacognitive strategies can be regarded as the behavioral output of cognitive control (Jansiewicz, 2008), cognitive control might play an important role in top-down listening processing. Cognitive inhibition and cognitive flexibility are two different yet correlated abilities of cognitive control (Miyake et al., 2000). They have also been considered as two critical abilities for L2 processing (e.g., Kieffer et al., 2013; Nouwens et al., 2016; Chang, 2020).

Cognitive inhibition is an active process of resisting extraneous or unwanted information that competes for neural resources due to the lack of sufficient capacity (Harnishfeger, 1995). During the process of SWS, irrelevantly activated lexical candidates must be inhibited by listeners to resolve lexical competition, thereby achieving correct and accurate listening processing (Norris et al., 1995). Bilingualism refers to the state of commanding two languages (Wilson and Mihalicek, 2011). According to the definition of bilingualism, bilinguals can be generally distinguished into balanced and unbalanced ones. The balanced bilinguals are those who acquired two languages simultaneously in their early childhood and can use both of their languages fluently. Unbalanced bilinguals are those who acquired their second language in their late childhood or adulthood without reaching the native-like level of proficiency (Vega-Mendoza et al., 2015). Studies have shown that no matter the types of bilingualism, both of the bilinguals’ languages would be activated during lexical processing (Sunderman and Kroll, 2006). Therefore, bilinguals have to inhibit lexical competition both within- and cross-language when segmenting L2 speech. And this continuous practice may enhance L2 learners’ ability of cognitive inhibition (Carlson and Meltzoff, 2008). These findings suggest that SWS may play a positive role in cognitive inhibition for L2 learners. In addition, previous literature has found that inhibitory control has a direct positive impact on L2 listening comprehension (Kim and Phillips, 2014), indicating that the ability to suppress irrelevant and competing stimuli is necessary for language learners to achieve the success of L2 listening processing.

Cognitive flexibility refers to the ability to shift perspectives, attention, and thinking flexibly based on changed circumstances (Diamond, 2013). During the process of SWS, listeners may exploit cognitive control to revise their miscomprehension of sentences (Novick et al., 2005), which would be caused by the activation of multiple and conflicting candidate representations (Weiss et al., 2009). Therefore, the demand for recurrent conflict monitoring and resolution in bilingual language processing is considered the likely source of bilinguals’ cognitive advantage (e.g., Bialystok and Shapero, 2005; Kroll et al., 2012). These cognitive advantages of bilinguals may reflect increased cognitive flexibility (Teubner-Rhodes et al., 2016). These findings altogether imply a positive influence of SWS on cognitive flexibility for L2 learners. Moreover, in the study of L2 learners’ behavioral strategy use in L2 listening, Murphy (1985) pointed out that the more proficient L2 listeners are more open and flexible, proved by their greater amount and more significant flexibility of strategy use in L2 listening. In line with the previous finding, Bacon (1992) found that L2 listeners’ success in L2 listening appears to be related to the total use of various strategies and the flexibility in changing strategies. On the other hand, studies in L2 learners’ underlying cognitive mechanisms have shown that cognitive flexibility and cognitive inhibition are, to some extent, correlated constructs (Miyake et al., 2000). Therefore, it is possible that cognitive flexibility may also be positively correlated with L2 listening processing.

It has been well-established that cognitive inhibition and cognitive flexibility function concurrently within L2 processing (e.g., Kieffer et al., 2013; Nouwens et al., 2016; Chang, 2020). During L2 listening processing, L2 learners may form multiple representations to predict the meaning of the utterance. However, the early prediction of the utterance may sometimes conflict with the later-arriving new information (Teubner-Rhodes et al., 2016). Therefore, it calls for the L2 learners to inhibit the prepotent and irrelevant representations and then revise their misinterpretations flexibly. Evidence has shown that cognitive flexibility is built on cognitive inhibition (Diamond, 2013).

The top-down model deciphers the mystery of listeners with a high level of L2 proficiency who comprehend utterances by flexibly applying heterogeneous metacognitive and cognitive strategies (Vandergrift, 2003), whereas falling short of providing sufficient evidence to unravel the approaches frequently used by less skilled listeners. Similar results have shown that top-down processes are more important for L2 learners with high proficiency than those with low proficiency (e.g., Li et al., 2020; Yang et al., 2021). However, L2 listening comprehension may fail if only the top-down process is initiated (Carrell and Eisterhold, 1983).

Therefore, the nature and defects of the aforementioned L2 listening models call for the systematic integration of both the bottom-up and the top-down models, i.e., the interactive model of L2 listening processing (Oxford, 1993; Rubin, 1994; Lynch, 1998, 2002; Mendelsohn, 1998). Vandergrift (2011) has claimed that the bottom-up and the top-down processes of L2 listening come into play together with each other and function independently. Orii-Akita (2014) found that the interactive model of L2 listening processing was more efficient than the pure bottom-up or top-down model in Japanese EFL university students. Even though very few studies have provided empirical evidence for the interactive model of L2 listening processing, the underlying cognitive mechanisms of this model remain largely unknown. Especially, there has been no empirical research investigating the roles of both cognitive inhibition and cognitive flexibility as top-down processes within the interactive model of L2 listening processing.

### The Current Study

**FIGURE 1:**
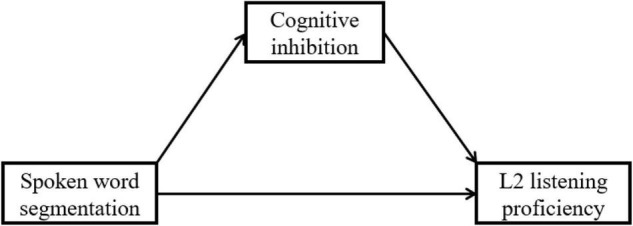
The hypothesized mediation role of cognitive inhibition.

From the literature mentioned above, it can be concluded that SWS may play a positive role in L2 listening, cognitive inhibition, and cognitive flexibility. Additionally, previous studies have suggested that cognitive inhibition and cognitive flexibility may positively predict L2 listening as two different yet correlated variables. Furthermore, studies have highlighted an interactive model that integrates both the bottom-up and top-down processing during L2 listening. The present study aims to provide further empirical evidence for the interactive model of L2 listening processing. We hypothesized the following:

**H1**: Cognitive inhibition mediates the relationship between L2 learners’ SWS and L2 listening proficiency (Figure 1).

**FIGURE 2:**
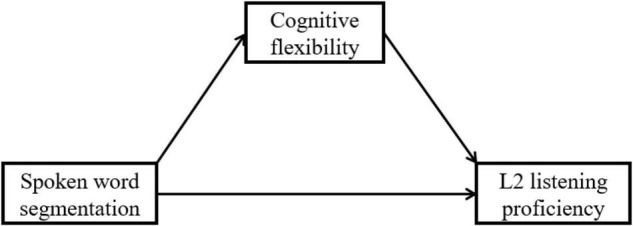
The hypothesized mediation role of cognitive flexibility.

**H2**: Cognitive flexibility mediates the relationship between L2 learners’ SWS and L2 listening proficiency (Figure 2).

## Methods

### Participants

One hundred and seventeen healthy volunteers joined the current study (26 males, 91 females, mean age: 19.38 ± 0.69 years). The participants were all Mandarin Chinese (L1) university students. English was reported as their L2. They were all unbalanced bilinguals who had acquired their L2 in their late childhood or adulthood. They all passed CET-4 (College English Test-Band 4), a national standardized English proficiency test for college students in China. The CET-4 lasts 125 min and measures test takers’ comprehensive English abilities. The test vocabulary covers about 4,500 English words. Participants’ language level of CET-4 ensures that they had qualified English language proficiency to do the language experiments in the current study, i.e., SWS and IELTS listening test. According to participants’ self-report, they were all right-handed and had no neurological and psychiatric disorders or substance abuse. They had the normal or corrected-to-normal vision and normal hearing.

### Measures

#### Spoken Word Segmentation

We adopted Cutler and Norris (1988)’s word-spotting paradigm to assess participants’ performance in English SWS using E-Prime 2.0 (Psychology Software Tools, Inc., Pittsburgh, PA, United States). We used a total of 96 stimuli of multisyllabic word strings (i.e., word plus nonsense syllable, such as *westej*, *lencool*) that consisted of real words (target word) and nonsense strings from Cutler and Shanley (2010) and Farrell (2015).

**FIGURE 3:**
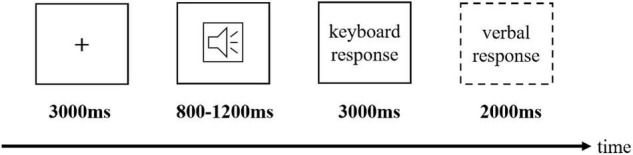
Experiment procedure of spoken word segmentation.

In a word-spotting task, participants would see a fixation cross on display for 8 s (Figure 3). They then would hear an audio stimulus (approximate duration 800-1,200 ms) played by two loudspeakers. Next, the participants had 3 s to identify the target word they had just heard in the audio stimulus. If they recognized the target word, they were required to make a keyboard response so that an additional 2 s would be given to them to speak out the target word (i.e., verbal response), e.g., speaking out English word *food* and *arm* in response to *foodeeb* and *armlek*. However, a new trial would start if the participant did not identify the target word in the audio stimulus within the time limit. We used a digital audio recording pen to record participants’ verbal responses, which were assessed after the experiment.

Several manipulations were performed to ensure the validity and reliability of the SWS test in the current participant sample. First, stimuli with target words in CET-4 test vocabulary were chosen to ensure that participants were familiar with target words. Second, target words of stimuli were 69 monosyllabic words and 27 multisyllabic words since Gitt (2006) suggested that modern English has 71.5% monosyllabic and 28.5% multisyllabic words. Third, the position of target words in stimuli was balanced. Half of the target words were in the initial position (e.g., *west* in *westej*). The other half were in the final position of the stimuli (e.g., *cool* in *lencool*). Fourth, we set two syllable boundary conditions (i.e., easy and difficult task conditions) following Cutler and Shanley (2010) and Farrell (2015). An easy task condition has unambiguous and easy to be identified word boundary, e.g., *dog* in *fubdog* and *arm* in *armlek*. In contrast, a difficult task condition has ambiguous and liaison word boundary, e.g., *agree* in *veamagree* and *food* in *foodeeb*. Fifth, we invited a female native speaker of American English to record the audio stimuli using a digital audio recording pen (44.1 kHz, 16 bit, mono). She was required to read in a continuous sequence and at a normal speed. We further used Cool Edit Pro 2.1 (Adobe Inc., San Jose, CA, United States) and Praat 6.1.04 (Boersma and Weenink, 2019) to process the audio stimuli.

#### Cognitive Inhibition

A Stroop color-word-interference task (hereafter Stroop task) was used to measure participants’ cognitive inhibition in E-Prime 2.0 (e.g., Stroop, 1935; Miyake et al., 2000). The paradigm is a widely accepted and classical experimental test (e.g., Vendrell et al., 1995; Heidlmayr et al., 2014). In each trial, one of the four color words was displayed on the screen in its Chinese character of “red,” “yellow,” “blue,” or “green” during the process of the task. These words were randomly presented either in a congruent or incongruent form with the colors red, yellow, blue, or green. Therefore, the color of the words was not matching the meaning of the words in some trials. At the beginning of each trial, a fixation cross was shown in the middle of the screen for 500 ms. Participants were then asked to identify the right color (instead of the meaning) of the stimulus words presented to them and to give their response by pressing the corresponding keys within a 2-second time limit. If they failed to respond within the time limit, a new trial would automatically follow. The Stroop task was conducted in Mandarin Chinese (i.e., participants’ L1) to avoid the influence of participants’ varied L2 competence and performance. Previous research has shown that the Chinese version of the Stroop task shares the same validity and reliability as its original version (Lee and Chan, 2000). In the present study, the Stroop task has 60 trials.

#### Cognitive Flexibility

Participants’ cognitive flexibility was measured by the cognitive flexibility inventory (CFI; Dennis and Vander Wal, 2010). It was developed as a brief self-report measure of the cognitive flexibility necessary for individuals to successfully challenge and replace inappropriate thoughts with more balanced and appropriate thinking. The CFI consists of 20 items and is distributed on a 5-point Likert scale ranging from 1 (“never”) to 5 (“very often”). The performance of participants’ cognitive flexibility is indicated by the sum of the 20 items of the CFI. It has been well established in the literature that the CFI has sufficient reliability and validity for measuring cognitive flexibility (Cronbach’s *α* = 0.91; Dennis and Vander Wal, 2010). It is also confirmed that the Chinese version of the CFI has satisfying reliability and validity as its English version (Cronbach’s *α* = 0.83; Wang et al., 2016). Therefore, the scale’s Chinese version was used in the present study.

#### Second Language Listening Proficiency

A listening test from *Cambridge English IELTS 9* (2013) assessed participants’ L2 listening proficiency. IELTS (the International English Language Testing System) is an authentic and highly recognized English proficiency test. The IELTS listening proficiency test consists of 40 questions that are distributed in 4 sections. The questions ask the test takers to either choose the correct answers or fill in the blanks with no more than three words after listening to the test audio. The entire test took approximately 40 minutes. It was administered and scored by an experienced associate professor who strictly followed the test instructions and answer keys of the IELTS listening test (full score = 40). A question before the test showed that none of the participants had ever taken this test before.

### Procedure and Statistical Analysis

The participants completed a demographic survey, an SWS task, a cognitive inhibition test, a cognitive flexibility questionnaire, and an L2 listening proficiency test. The demographic survey and cognitive flexibility questionnaire were distributed via an online survey platform^1^. The SWS task and cognitive inhibition test were performed using E-Prime 2.0. The L2 listening proficiency test was a paper-based test that was completed in a quiet room.

IBM Statistical Package for Social Sciences (SPSS) version 25.0 (SPSS Inc., Chicago, United States) was used for the descriptive statistics and correlation analyses. The PROCESS (v. 3.5) macro for SPSS was used to test our hypotheses (Preacher and Hayes, 2004; Hayes, 2013). Model 6 was used to test the hypothesized mediation effects, with a bootstrapping sample size of 5,000 and 95% confidence intervals (CIs). We set SWS as the independent variable, cognitive inhibition and cognitive flexibility as the mediation variables, and L2 listening proficiency as the dependent variable.

## Results

### Descriptive Statistics and Correlation Analysis

Participants’ performance in SWS (52.872 ± 6.670), cognitive inhibition (46.359 ± 5.255), cognitive flexibility (64.641 ± 7.588), and L2 listening proficiency (27.410 ± 5.238) were tested. Table 1 shows the means of, standard deviations of, and correlations among those variables. SWS was in positive correlations with cognitive inhibition (*r* = 0.329), cognitive flexibility (*r* = 0.405), and L2 listening proficiency (*r* = 0.361). These results suggest that participants who performed better in SWS also had better performance in the cognitive inhibition, cognitive flexibility, and L2 listening tasks. Cognitive inhibition was in positive correlations with cognitive flexibility (*r* = 0.335) and L2 listening proficiency (*r* = 0.362), showing that participants who had better performance in cognitive inhibition tasks also showed greater ability of cognitive flexibility and higher L2 listening proficiency than their counterparts. Cognitive flexibility was positively correlated with L2 listening proficiency (*r* = 0.372), suggesting that participants who reported more advanced ability of cognitive flexibility also had higher L2 listening proficiency.

**TABLE 1:** Means, standard deviations, and correlations between variables.

Variables	*M* ± *SD*	1	2	3	4
1. SWS	52.872 ± 6.670	1			
2. Cognitive inhibition	46.359 ± 5.255	0.329	1		
3. Cognitive flexibility	64.641 ± 7.588	0.405	0.335	1	
4. L2 listening proficiency	27.410 ± 5.238	0.361	0.362	0.372	1

*SWS, spoken word segmentation.*

*All ps < 0.05.*

### Mediation Analysis

**FIGURE 4:**
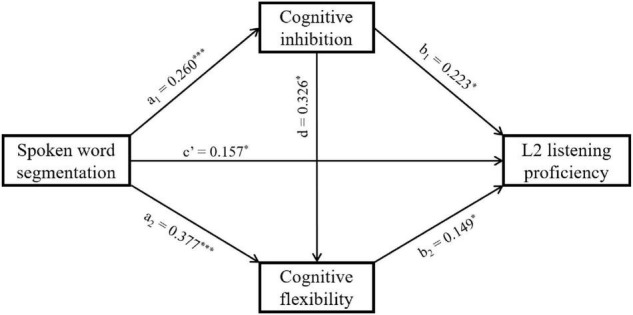
Mediation roles of cognitive inhibition and cognitive flexibility in the association between SWS and L2 listening proficiency. The depicted is the path diagram of the multiple mediation analysis using the PROCESS (Model 6) macro for SPSS. *p < 0.05, ^***^p < 0.001.

We used the PROCESS (v. 3.5) extension for SPSS version 25.0 for mediation analyses. The multiple mediation analysis was performed to test the role of cognitive inhibition and cognitive flexibility in the association between SWS and L2 listening proficiency. The mediation model was significant and accounted for a significant proportion of the variance in explaining the relationship between SWS and L2 listening proficiency [*R*^2^ = 0.233, *F* (3, 113) = 11.444, *p* < 0.001]. SWS had a positive influence on cognitive inhibition (*β* = 0.260, *SE* = 0.069, *p* < 0.001) and L2 listening proficiency (*β* = 0.157, *SE* = 0.073, *p* < 0.05). Meanwhile, cognitive inhibition had a positive influence on cognitive flexibility (*β* = 0.326, *SE* = 0.127, *p* < 0.05) and L2 listening proficiency (*β* = 0.223, *SE* = 0.089, *p* < 0.05). These results suggest that cognitive inhibition mediates the relationship between participants’ performance in SWS and L2 listening proficiency. Moreover, SWS also had a positive impact on cognitive flexibility (*β* = 0.377, *SE* = 0.100, *p* < 0.001). Additionally, cognitive flexibility had a positive influence on L2 listening proficiency (*β* = 0.149, *SE* = 0.064, *p* < 0.05). These results suggest that cognitive flexibility mediated the relationship between participants’ SWS performance and L2 listening proficiency. The results are shown in Figure 4.

Furthermore, SWS had two paths of indirect influence on L2 listening proficiency, i.e., through cognitive inhibition (*β* = 0.058, *SE* = 0.028, 95% CI = [0.010, 0.117]) and cognitive flexibility (*β* = 0.056, *SE* = 0.029, 95% CI = [0.009, 0.121]) respectively. However, the influence of SWS on L2 listening proficiency through sequential effects of cognitive inhibition and cognitive flexibility is not significant (*β* = 0.013, *SE* = 0.008, 95% CI = [–0.001, 0.030]), suggesting that the chain mediation model is invalid. These results are shown in Table 2.

**TABLE 2:** Direct and indirect effects of SWS on L2 listening proficiency.

	95% CI	Effect
**Direct effect**		
SWS → L2 listening proficiency	[0.013, 0.300]	0.157
**Indirect effect**		
SWS → cognitive inhibition → L2 listening proficiency	[0.010, 0.117]	0.058
SWS → cognitive flexibility → L2 listening proficiency	[0.009, 0.121]	0.056
SWS → cognitive inhibition → cognitive flexibility → L2 listening proficiency	[−0.001, 0.030]	0.013

*SWS, spoken word segmentation.*

## Discussion

Prior studies have suggested that non-native speakers are capable of using heterogenous segmentation cues in L2 listening (e.g., Sanders et al., 2002) and have emphasized the positive impact of SWS on the success of L2 listening processing (e.g., Field, 2003; Cutler and Shanley, 2010). Even though listening processing, as a complex cognitive activity, requires bottom-up linguistic skills, such as vocabulary, spoken word segmentation, and recognition, they cannot sufficiently account for the success of listening comprehension (Kim and Phillips, 2014). The current study provided empirical evidence for the interactive model of L2 listening processing by investigating the potential roles of cognitive inhibition and cognitive flexibility in the relationship between L2 learners’ English SWS and L2 listening proficiency. The results of our study showed that SWS was in positive correlations with cognitive inhibition and L2 listening proficiency. In addition, SWS also had a significantly positive influence on cognitive flexibility. Further mediation analyses revealed that both cognitive inhibition and cognitive flexibility mediated the relationship between participants’ SWS and L2 listening proficiency.

### Spoken Word Segmentation and Second Language Listening Proficiency

The current study showed that L2 listeners’ SWS might have a direct influence on bilinguals’ L2 listening proficiency. SWS is a crucial and challenging bottom-up process exploited by language learners during L2 listening (Vandergrift, 2011). Previous research has pointed out that language learners might make good use of various linguistic cues to segment speech stream into meaningful linguistic units during the process of L2 listening (Goyet et al., 2010). L2 learners gradually combine the segmented linguistic units into larger chunks of units (e.g., from the phoneme level to the discourse level) to achieve successful L2 listening (Vandergrift, 2011). Therefore, the result of the current study is within our expectation that L2 learners who have better performance in SWS also exhibit higher L2 listening proficiency.

### The Mediation Role of Cognitive Inhibition

The current study found that bilinguals’ SWS might have a positive effect on their cognitive inhibition. Previous studies have revealed that during the process of SWS, both of the two languages of bilinguals would be activated simultaneously for spoken language processing (e.g., Marian et al., 2003; Marian and Spivey, 2003a,b; Blumenfeld and Marian, 2007; Canseco-Gonzalez et al., 2010). This process would lead to the activation of multiple candidate words that compete for recognition (McQueen et al., 1994). Bilinguals thus may need to adopt a certain mechanism of language control to inhibit the lexical competition of both within-language and cross-language to deal with the irrelevantly activated candidates to achieve the success of L2 listening processing (Green, 1998).

The result of the current study is in line with previous findings that participants who perform better in SWS also have better performance in the tasks of cognitive inhibition (e.g., Blumenfeld and Marian, 2011; Mercier et al., 2014). A possible explanation is that bilinguals might have to deal with high degrees of lexical competition in their daily communication, presumably caused by the co-activation of their two languages, especially if the communication is in their L2 (e.g., Weber and Cutler, 2004; Canseco-Gonzalez et al., 2010). With the continuous practice of segmenting L2 speech stream and inhibiting distracting candidate words in their daily life, bilinguals might have better performance in cognitive inhibition, thereby exhibiting top-down cognitive advantages over their monolingual counterparts.

We found that cognitive inhibition was in positive correlation with L2 listening proficiency. Cognitive inhibition is one of the top-down cognitive control mechanisms. It was confirmed to be conducive to academic achievements, such as math (e.g., Best et al., 2011) and reading (e.g., Best et al., 2011; Cartwright, 2012; Nouwens et al., 2016, 2021). The finding of our study was convergent with the literature mentioned above in other disciplines and extended previous research by showing that cognitive inhibition would also predict the success of L2 listening. It suggests that L2 learners’ relatively more advanced cognitive inhibition ability than monolinguals would let them better resist the interference of irrelevant competing mental representation and candidate words. Moreover, L2 learners can inhibit the inclination to unconsciously apply their L1 segmenting procedures during L2 listening (Cutler, 2000; Cross, 2009). Therefore, another possible explanation would be that bilinguals who possess a greater ability of cognitive inhibition could perform better in suppressing the natural tendency of utilizing their L1 segmenting procedures for L2 listening processing. Such continuous inhibition might, in turn, lead to higher L2 listening proficiency.

The result of the current study indicated that in addition to the significant and direct impact of SWS on L2 listening, bilinguals’ SWS also had an indirect influence on their L2 listening proficiency through cognitive inhibition. Although previous literature has suggested that SWS is not the only factor for the success of L2 listening and cognitive inhibition may play a part in this process (e.g., McQueen et al., 1994; Norris, 1994; Norris et al., 1995; Luce and Cluff, 1998; Luce and Lyons, 1999), to the best of our knowledge, only a few studies have provided direct empirical evidence. Therefore, we extended this line of research by expanding the participant sample of research to bilinguals whose L2 is in Indo-European languages whereas their L1 is in Sino-Tibetan languages.

In accordance with our first hypothesis, the result of mediation analysis in the current study showed that cognitive inhibition mediated the relationship between participants’ SWS and L2 listening proficiency. In other words, L2 learners’ SWS might have a positive influence on their L2 listening proficiency through cognitive inhibition. Our results support previous findings that bilinguals’ language experience may enhance their ability of cognitive control (e.g., Xie and Dong, 2017). Therefore, by the continuous practice of segmenting L2 speech during language learners’ daily communication, they would possess a greater ability to inhibit linguistic competition both within-language and cross-language. Consequently, this more advanced ability of cognitive inhibition would then contribute to better performance in L2 listening processing. The results suggested that besides the bottom-up listening processing (i.e., SWS), L2 listeners also recruited top-down cognitive control (i.e., cognitive inhibition) to achieve successful L2 listening. Such results provided robust evidence for the interactive model of L2 listening processing.

### The Mediation Role of Cognitive Flexibility

As mentioned earlier, the literature has pointed out that the constructs of cognitive inhibition and cognitive flexibility are strongly intertwined and interdependent (Miyake et al., 2000; Chevalier and Blaye, 2008). Therefore, we expected that cognitive flexibility should also play a similar role in the relationship between SWS and L2 listening proficiency as cognitive inhibition does. However, cognitive flexibility and cognitive inhibition have still, to some extent, been considered as two distinct mechanisms (Miyake et al., 2000), and cognitive flexibility is thought to be built on cognitive inhibition (Diamond, 2013). Therefore, it is evident that the enhancement of one aspect of the two abilities does not necessarily mean the facilitation of the other. The exact role played by cognitive flexibility in the relationship between SWS and L2 listening proficiency remains to be confirmed.

The present study extended previous research by finding that bilinguals’ SWS might have a positive influence on their cognitive flexibility. Previous research has found that even though it might be inconducive for successful L2 listening, language learners are seemingly reluctant to abandon the inappropriate SWS procedure they have built for L2 listening comprehension (Field, 2008). This finding stressed the importance of cognitive flexibility during the process of SWS and could serve as indirect evidence for our result. In addition, prior research has shown that when confronted with L2 input, language learners would have multiple interpretations of the utterance (Weiss et al., 2009). Given the ephemeral and multifaceted nature of real-world communication, L2 listeners must process the speech that they have just heard while simultaneously receiving new upcoming utterances by the interlocutors during the bottom-up listening processing (i.e., SWS). This exact nature of communication calls for the L2 listeners’ ability of cognitive flexibility to actively shift their focus and mental state not only between the meaning of the words they have just heard and the new input yet to come (Vandergrift, 2011) but also the co-activated irrelevant candidate words and interpretations between the two languages that they know (Dong and Xie, 2014). The result of our study is consistent with previous findings by showing that L2 listeners may use cognitive flexibility to consciously shift between languages and revise misinterpretations triggered by competing alternatives (e.g., Novick et al., 2005; Ye and Zhou, 2009). Therefore, this continuous demand of segmenting L2 speech and flexibly switching between two languages and multiple words during communication reflects better cognitive flexibility for language learners as an outcome.

As mentioned above, cognitive flexibility is considered to be built on cognitive inhibition (Diamond, 2013) since the shifting of perspectives, thinking, and attention requires the suppression of irrelevant or prepotent cues (Bialystok, 2015). Therefore, the results identified in the current study also suggested that cognitive flexibility was positively associated with L2 listening proficiency, similar to the role of cognitive inhibition as we expected before the present study. Previous studies have shown that cognitive flexibility has a significant influence on the decoding and language skills of children (e.g., Cartwright, 2012; Kieffer et al., 2013), young adolescents (e.g., Ober et al., 2019), and adults (e.g., Follmer and Sperling, 2019). The current study’s finding is partially consistent with preceding research by suggesting that the L2 listeners who perform better in switching among alternative words and interpretations would exhibit greater language skills in the tasks of L2 listening, thereby showing higher L2 listening proficiency.

In line with the second hypothesis, the result of the current study showed that cognitive flexibility mediated the relationship between participants’ SWS and L2 listening proficiency. The result provided empirical evidence to further verify that cognitive inhibition is not the only top-down control mechanism that would contribute to L2 listening. We found in our study that, besides the direct impact, there was an indirect influence of L2 learners’ SWS on their L2 listening proficiency through cognitive flexibility. A possible explanation for this finding is that by consistently resolving linguistic competitions during the process of SWS in bilinguals’ everyday life, their ability of cognitive flexibility may be facilitated (Bialystok, 2005) and contribute to the performance in L2 listening.

### The Interactive Model of Second Language Listening

The current study further extended the research by examining the interaction of cognitive inhibition and cognitive flexibility in the relationship between SWS and L2 listening proficiency. Studies have previously found that the demands of resolving and processing linguistic competitions for L2 learners are the likely source of bilinguals’ cognitive advantages over monolinguals (e.g., Bialystok, 2005; Kroll et al., 2012). In addition, such an increased cognitive control mechanism was positively related to L2 listening comprehension (e.g., Kim and Phillips, 2014). A reasonable interpretation is that bilinguals might possess better overall cognitive control due to their continuous engagement in competitive solutions among candidate words both within-language and cross-language during the process of SWS.

Moreover, our study also extended preceding research by showing that, besides the bottom-up listening processing of using linguistic cues (i.e., SWS), non-linguistic top-down cognitive processes such as cognitive inhibition and cognitive flexibility are also recruited by L2 listeners in L2 listening processing. At least on the lexical level, such findings provided further empirical evidence for the interactive model of L2 listening processing. Studies have shown that participants who use top-down listening processes can manage their attentional resources to achieve better L2 listening processing (e.g., Oh and Lee, 2014). In line with previous findings, the result of our study suggests that successful L2 listening not only requires the listeners to segment aural texts into meaningful and proper words during communication but also requires the co-activation of top-down cognitive control mechanisms to inhibit irrelevant competing candidate words actively and flexibly shift among words and interpretations.

### Limitations

Several limitations in the present study, as well as suggestions for future studies, should be noted. First, the participants were all healthy Chinese young adults of similar ages, and most of them were females. Future studies should be conducted with a more balanced gender distribution and a more diverse participant group. Second, given that the components of cognitive control are still under heated debate, the current study conducted behavioral experiments only on cognitive inhibition and cognitive flexibility. Future studies should consider taking the influence of other top-down cognitive control mechanisms on L2 listening into account, such as working memory. Third, it is worth notifying that the relationship between bottom-up and top-down listening processing was not fully explored. Future studies should further investigate in what proportion listeners preferentially recruit the bottom-up and top-down processing of L2 listening. Fourth, the data collected in the current study was synchronic. The data may not fully account for the causal relationships among the variables. Future studies should conduct longitudinal investigations to establish causal relationships among bilinguals’ SWS, cognitive control, and L2 listening proficiency.

## Conclusion

The current study showed that L2 listening proficiency was in positive correlations with SWS, cognitive inhibition, and cognitive flexibility ability. The current study also investigated cognitive mechanisms underlying the process of SWS. The mediation analyses revealed that both cognitive inhibition and cognitive flexibility mediate the relationship between L2 learners’ SWS and L2 listening proficiency. The results suggested that, along with the bottom-up listening processing of SWS, L2 listeners also exploited top-down cognitive control mechanisms to inhibit irrelevant competing candidate words and shift among words and interpretations to achieve successful L2 listening processing. The findings provided further empirical evidence for the interactive model of L2 listening processing.

The findings of our study may have important implications for future research on L2 listening to investigate the influence of non-linguistic cognitive control mechanisms during L2 listening processing. Furthermore, the findings of our study may also have implications for future L2 teaching and learning. L2 learners should attach importance to the inseparable contribution of both bottom-up and top-down processes in L2 listening and actively put them into practice. For future instructional methodologies, L2 teachers should also consider the interactive model of L2 listening processing to design more comprehensive curricula for language learners from both linguistic and cognitive perspectives.

## Data Availability Statement

The raw data supporting the conclusions of this article will be made available by the authors, without undue reservation.

## Ethics Statement

The studies involving human participants were reviewed and approved by the Academic Committee of the Ministry of Education Key Laboratory of Modern Teaching Technology of Shaanxi Normal University. The patients/participants provided their written informed consent to participate in this study.

## Author Contributions

YY designed the study, finished the experiments and surveys, performed the data analysis, and drafted the manuscript. GY drafted and revised the manuscript. YL collected data, reviewed and revised the manuscript, and supervised the study. All authors contributed to the article and approved the submitted version.

## Conflict of Interest

The authors declare that the research was conducted in the absence of any commercial or financial relationships that could be construed as a potential conflict of interest.

## Publisher’s Note

All claims expressed in this article are solely those of the authors and do not necessarily represent those of their affiliated organizations, or those of the publisher, the editors and the reviewers. Any product that may be evaluated in this article, or claim that may be made by its manufacturer, is not guaranteed or endorsed by the publisher.

## Footnotes

1
http://www.wjx.cn

## References

[B1] Bacon S. M. (1992). Phases of listening to authentic input in Spanish: a descriptive study. *Foreign Lang. Ann.* 25 317–333. 10.1111/j.1944-9720.1992.tb00552.x

[B2] Best J. R. Miller P. H. Naglieri J. A. (2011). Relations between executive function and academic achievement from ages 5 to 17 in a large, representative national sample. *Learn. Individ. Differ.* 21 327–336. 21845021 10.1016/j.lindif.2011.01.007 PMC3155246

[B3] Bialystok E. (2005). “Consequences of bilingualism for cognitive development,” in *Handbook of Bilingualism: Psycholinguistic Approaches*, eds Kroll J. F. de Groot A. M. B. (Oxford: Oxford University Press), 417–432.

[B4] Bialystok E. (2015). Bilingualism and the development of executive function: the role of attention. *Child Dev. Perspect.* 9 117–121. 10.1111/cdep.12116 26019718 PMC4442091

[B5] Bialystok E. Shapero D. (2005). Ambiguous benefits: the effect of bilingualism on reversing ambiguous figures. *Dev. Sci.* 8 595–604. 10.1111/j.1467-7687.2005.00451.x 16246250

[B6] Blumenfeld H. K. Marian V. (2007). Constraints on parallel activation in bilingual spoken language processing: examining proficiency and lexical status using eye-tracking. *Lang. Cogn. Process.* 22 633–660. 10.1080/01690960601000746

[B7] Blumenfeld H. K. Marian V. (2011). Bilingualism influences inhibitory control in auditory comprehension. *Cognition* 118 245–257. 10.1016/j.cognition.2010.10.012 21159332 PMC3582323

[B8] Boersma P. Weenink D. (2019). *Praat: Doing Phonetics by Computer In (Version 6.1.04)*. Available online at: https://eric.ed.gov/?id=ED278275 (accessed August 14, 2019).

[B9] Canseco-Gonzalez E. Brehm L. Brick C. A. Brown-Schmidt S. Fischer K. Wagner K. (2010). Carpet or Cárcel: the effect of age of acquisition and language mode on bilingual lexical access. *Lang. Cogn. Process.* 25 669–705. 10.1080/01690960903474912

[B10] Carlson S. M. Meltzoff A. N. (2008). Bilingual experience and executive functioning in young children. *Dev. Sci.* 11 282–298. 10.1111/j.1467-7687.2008.00675.x 18333982 PMC3647884

[B11] Carrell P. L. Eisterhold J. C. (1983). Schema theory and ESL reading pedagogy. *TESOL Q.* 17 553–573. 10.2307/3586613

[B12] Cartwright K. B. (2012). Insights from cognitive neuroscience: the importance of executive function for early reading development and education. *Early Educ. Dev.* 23 24–36. 10.1080/10409289.2011.615025

[B13] Chang I. (2020). Influences of executive function, language comprehension, and fluency on young children’s reading comprehension. *J. Early Childh. Res.* 18 44–57. 10.1177/1476718X19875768

[B14] Chevalier N. Blaye A. (2008). Cognitive flexibility in preschoolers: the role of representation activation and maintenance. *Dev. Sci.* 11 339–353. 10.1111/j.1467-7687.2008.00679.x 18466368

[B15] Cole R. A. Jakimik J. (1980). How are syllables used to recognize words? *J. Acoust. Soc. Am.* 67 965–970. 10.1121/1.383939 7358921

[B16] Cross J. (2009). Diagnosing the process, text, and intrusion problems responsible for L2 listeners’ decoding errors. *Asian EFL J.* 11 31–53.

[B17] Cutler A. (2000). Listening to a second language through the ears of a first. *Interpreting* 5 1–23. 10.1075/intp.5.1.02cut 33486653

[B18] Cutler A. Norris D. (1988). The role of strong syllables in segmentation for lexical access. *J. Exp. Psychol. Hum. Percept. Perform.* 14 113–121. 10.1037/0096-1523.14.1.113

[B19] Cutler A. Shanley J. (2010). “Validation of a training method for L2 continuous-speech segmentation,” in *Proceedings of the 11th Annual Conference of the International Speech Communication Association (Interspeech 2010)*, Makuhari.

[B20] Dennis J. P. Vander Wal J. S. (2010). The cognitive flexibility inventory: instrument development and estimates of reliability and validity. *Cogn. Ther. Res.* 34 241–253. 10.1007/s10608-009-9276-4

[B21] Diamond A. (2013). Executive functions. *Annu. Rev. Psychol.* 64 135–168. 10.1146/annurev-psych-113011-143750 23020641 PMC4084861

[B22] Dong Y. Xie Z. (2014). Contributions of second language proficiency and interpreting experience to cognitive control differences among young adult bilinguals. *J. Cogn. Psychol.* 26 506–519. 10.1080/20445911.2014.924951

[B23] Farrell J. (2015). *Training L2 Speech Segmentation with Word-Spotting.* Sydney, NSW: Western Sydney University.

[B24] Feyten C. M. (1991). The power of listening ability: an overlooked dimension in language acquisition. *Mod. Lang. J.* 75 173–180. 10.2307/328825

[B25] Field J. (2003). Promoting perception: lexical segmentation in L2 listening. *ELT J.* 57 325–334. 10.1093/elt/57.4.325

[B26] Field J. (2008). Revising segmentation hypotheses in first and second language listening. *System* 36 35–51. 10.1016/j.system.2007.10.003

[B27] Flowerdew J. Miller L. (2005). *Second Language Listening: Theory and Practice.* Stuttgart: Ernst Klett Sprachen.

[B28] Follmer D. J. Sperling R. A. (2019). A latent variable analysis of the contribution of executive function to adult readers’ comprehension of science text: the roles of vocabulary ability and level of comprehension. *Read. Writ.* 32 377–403. 10.1007/s11145-018-9872-3

[B29] Fotos S. (2001). “Cognitive approaches to grammar instruction,” in *Teaching English as A Second or Foreign Language*, Vol. 3 ed. Celce-Murcia M. (Boston, MA: Heinle and Heinle), 267–283.

[B30] Gitt W. (2006). *In the Beginning was Information: A Scientist Explains the Incredible Design in Nature.* Green Forest, AR: New Leaf Publishing Group.

[B31] Goh C. C. M. (2000). A cognitive perspective on language learners’ listening comprehension problems. *System* 28 55–75. 10.1016/s0346-251x(99)00060-3

[B32] Goyet L. de Schonen S. Nazzi T. (2010). Words and syllables in fluent speech segmentation by French-learning infants: an ERP study. *Brain Res.* 1332 75–89. 10.1016/j.brainres.2010.03.047 20331982

[B33] Graham S. (2006). Listening comprehension: the learners’ perspective. *System* 34 165–182. 10.1016/j.system.2005.11.001

[B34] Green D. W. (1998). Mental control of the bilingual lexico-semantic system. *Biling. Lang. Cogn.* 1 67–81. 10.1017/S1366728998000133

[B35] Harnishfeger K. K. (1995). “The development of cognitive inhibition: theories, definitions, and research evidence,” in *Interference and Inhibition in Cognition*, eds Dempster F. N. Brainerd C. J. (Amsterdam: Elsevier), 175–204.

[B36] Hayes A. F. (2013). *Introduction to Mediation, Moderation, and Conditional Process Analysis: A Regression-Based Approach.* New York, NY: The Guilford Press, 10.1111/jedm.12050

[B37] Heidlmayr K. Moutier S. Hemforth B. Courtin C. Tanzmeister R. Isel F. (2014). Successive bilingualism and executive functions: the effect of second language use on inhibitory control in a behavioural Stroop Colour Word task. *Biling. Lang. Cogn.* 17 630–645. 10.1017/S1366728913000539

[B38] Hinkel E. (2006). Current perspectives on teaching the four skills. *TESOL Q.* 40 109–131. 10.2307/40264513

[B39] Jansiewicz E. M. (2008). *The relationship Between Executive Functions and Metacognitive Strategy Learning and Application.* Doctoral dissertation. Atlanta, GA: Georgia State University.

[B40] Kieffer M. J. Vukovic R. K. Berry D. (2013). Roles of attention shifting and inhibitory control in fourth-grade reading comprehension. *Read. Res. Q.* 48 333–348. 10.1002/rrq.54

[B41] Kim Y.-S. Phillips B. (2014). Cognitive correlates of listening comprehension. *Read. Res. Q.* 49 269–281. 10.1002/rrq.74

[B42] Kroll J. F. Dussias P. E. Bogulski C. A. Kroff J. R. V. (2012). “Juggling two languages in one mind: what bilinguals tell us about language processing and its consequences for cognition,” in *Psychology of Learning and Motivation*, Vol. 56 ed. Ross B. H. (Amsterdam: Elsevier), 229–262.

[B43] Lee T. M. Chan C. C. (2000). Stroop interference in Chinese and English. *J. Clin. Exp. Neuropsychol.* 22 465–471. 10.1076/1380-3395(200008)22:4;1-0;FT465 10923056

[B44] Li Y. Yang Y. Tang A. C. Liu N. Wang X. Du Y. (2020). English spoken word segmentation activates the prefrontal cortex and temporo-parietal junction in Chinese ESL learners: a functional near-infrared spectroscopy (fNIRS) study. *Brain Res.* 1733:146693. 10.1016/j.brainres.2020.146693 32006554

[B45] Luce P. A. Cluff M. S. (1998). Delayed commitment in spoken word recognition: evidence from cross-modal priming. *Percept. Psychophys.* 60 484–490. 10.3758/BF03206868 9599997

[B46] Luce P. A. Lyons E. A. (1999). Processing lexically embedded spoken words. *J. Exp. Psychol. Hum. Percept. Perform.* 25 174–183. 10.1037//0096-1523.25.1.174 10069031

[B47] Lynch T. (1998). Theoretical perspectives on listening. *Annu. Rev. Appl. Linguist.* 18 3–19. 10.1017/S0267190500003457

[B48] Lynch T. (2002). *Listening: Questions of level.* Oxford: Oxford University Press.

[B49] Marian V. Spivey M. (2003a). Bilingual and monolingual processing of competing lexical items. *Appl. Psycholinguist.* 24 173–193. 10.1017/S0142716403000092

[B50] Marian V. Spivey M. (2003b). Competing activation in bilingual language processing: within-and between-language competition. *Biling. Lang. Cogn.* 6 97–115. 10.1017/S1366728903001068

[B51] Marian V. Spivey M. Hirsch J. (2003). Shared and separate systems in bilingual language processing: converging evidence from eyetracking and brain imaging. *Brain Lang.* 86 70–82. 10.1016/S0093-934X(02)00535-7 12821416

[B52] McQueen J. M. Norris D. Cutler A. (1994). Competition in spoken word recognition: spotting words in other words. *J. Exp. Psychol. Learn. Mem. Cogn.* 20 621–638. 10.1037/0278-7393.20.3.621 8744962

[B53] Mendelsohn D. J. (1998). Teaching listening. *Annu. Rev. Appl. Linguist.* 18 81–101. 10.1017/S0267190500003494

[B54] Mercier J. Pivneva I. Titone D. (2014). Individual differences in inhibitory control relate to bilingual spoken word processing. *Biling. Lang. Cogn.* 17 89–117. 10.1017/S1366728913000084

[B55] Miyake A. Friedman N. P. Emerson M. J. Witzki A. H. Howerter A. Wager T. D. (2000). The unity and diversity of executive functions and their contributions to complex :“Frontal Lobe” tasks: a latent variable analysis. *Cogn. Psychol.* 41 49–100. 10.1006/cogp.1999.0734 10945922

[B56] Murphy J. M. (1985). *An Investigation into the Listening Strategies of ESL College Students.* Available online at: https://eric.ed.gov/?id=ED278275 (accessed November 10, 2021).

[B57] Murphy J. M. (1991). Oral communication in TESOL: integrating speaking, listening, and pronunciation. *TESOL Q.* 25 51–75. 10.2307/3587028

[B58] Namaziandost E. Neisi L. Mahdavirad F. Nasri M. (2019). The relationship between listening comprehension problems and strategy usage among advance EFL learners. *Cogent Psychol.* 6:1691338. 10.1080/23311908.2019.1691338

[B59] Norris D. (1994). Shortlist: a connectionist model of continuous speech recognition. *Cognition* 52 189–234. 10.1016/0010-0277(94)90043-4

[B60] Norris D. McQueen J. M. Cutler A. (1995). Competition and segmentation in spoken-word recognition. *Journal of Exp. Psychol. Learn. Mem. Cogn.* 21 1209–1228. 10.1037/0278-7393.21.5.1209 8744962

[B61] Nouwens S. Groen M. A. Verhoeven L. (2016). How storage and executive functions contribute to children’s reading comprehension. *Learn. Individ. Differ.* 47 96–102. 10.1016/j.lindif.2015.12.008

[B62] Nouwens S. Groen M. A. Kleemans T. Verhoeven L. (2021). How executive functions contribute to reading comprehension. *Br. J. Educ. Psychol.* 91 169–192. 10.1111/bjep.12355 32441782 PMC7983997

[B63] Novick J. M. Trueswell J. C. Thompson-Schill S. L. (2005). Cognitive control and parsing: reexamining the role of Broca’s area in sentence comprehension. *Cogn. Affect. Behav. Neurosci.* 5 263–281. 10.3758/CABN.5.3.263 16396089

[B64] Ober T. M. Brooks P. J. Plass J. L. Homer B. D. (2019). Distinguishing direct and indirect effects of executive functions on reading comprehension in adolescents. *Read. Psychol.* 40 551–581. 10.1080/02702711.2019.1635239

[B65] Oh E. Lee C. M. (2014). The role of linguistic knowledge and listening strategies in bottom-up and top-down processing of L2 listening. *English Teach.* 69 149–173. 10.15858/engtea.69.2.201406.149

[B66] Orii-Akita M. (2014). “The effectiveness of interactive teaching methods in EFL classrooms: a comparison with bottom-up and top-down methods,” in *Proceedings of the International Symposium on the Acquisition of Second Language Speech, Concordia working Papers in Applied Linguistics*, Vol. 5 464–477. Available online at: doe.concordia.ca/copal/documents32_OriiAkita_Vol5.pdf (accessed November 8, 2021).

[B67] Oxford R. L. (1993). Research update on teaching L2 listening. *System* 21 205–211. 10.1016/0346-251X(93)90042-F

[B68] Preacher K. J. Hayes A. F. (2004). SPSS and SAS procedures for estimating indirect effects in simple mediation models. *Methods Instrum. Comput.* 36 717–731. 10.3758/BF03206553 15641418

[B69] Rubin J. (1994). A review of second language listening comprehension research. *Mod. Lang. J.* 78 199–221. 10.2307/329010

[B70] Sanders L. D. Neville H. J. Woldorff M. G. (2002). Speech segmentation by native and non-native speakers. *J. Speech Lang. Hear. Res.* 45 519–530. 10.1044/1092-4388(2002/041) 12069004 PMC2532534

[B71] Snow M. A. (2005). “A model of academic literacy for integrated language and content instruction,” in *Handbook of Research in Second Language Teaching and Learning*, ed. Hinkel E. (London: Routledge), 717–736. 10.4324/9781410612700-52

[B72] Stroop J. R. (1935). Studies of interference in serial verbal reactions. *J. Exp. Psychol.* 18 643–662. 10.1037/h0054651

[B73] Sunderman G. Kroll J. F. (2006). First language activation during second language lexical processing: an investigation of lexical form, meaning, and grammatical class. *Stud. Sec. Lang. Acquis.* 28 387–422. 10.1017/S0272263106060177

[B74] Teubner-Rhodes S. E. Mishler A. Corbett R. Andreu L. Sanz-Torrent M. Trueswell J. C. (2016). The effects of bilingualism on conflict monitoring, cognitive control, and garden-path recovery. *Cognition* 150 213–231. 10.1016/j.cognition.2016.02.011 26918741

[B75] Vandergrift L. (1997). The comprehension strategies of second language (French) listeners: a descriptive study. *Foreign Lang. Ann.* 30 387–409. 10.1111/j.1944-9720.1997.tb02362.x

[B76] Vandergrift L. (2003). Orchestrating strategy use: toward a model of the skilled second language listener. *Lang. Learn.* 53 463–496. 10.1111/1467-9922.00232

[B77] Vandergrift L. (2007). Recent developments in second and foreign language listening comprehension research. *Lang. Teach.* 40 191–210. 10.1017/S0261444807004338

[B78] Vandergrift L. (2008). “Learning strategies for listening comprehension,” in *Language Learning Strategies in Independent Settings*, eds Hurd S. Lewis T. (Bristol: Multilingual Matters), 84–102. 10.21832/9781847690999-007

[B79] Vandergrift L. (2011). “Second language listening,” in *Handbook of Research in Second Language Teaching and Learning*, Vol. 2 ed. Hinkel E. (London: Routledge), 455–471.

[B80] Vega-Mendoza M. West H. Sorace A. Bak T. H. (2015). The impact of late, non-balanced bilingualism on cognitive performance. *Cognition* 137 40–46. 10.1016/j.cognition.2014.12.008 25596355

[B81] Vendrell P. Junqué C. Pujol J. Jurado M. A. Molet J. Grafman J. (1995). The role of prefrontal regions in the Stroop task. *Neuropsychologia* 33 341–352. 10.1016/0028-3932(94)00116-7 7792000

[B82] Wang Y. Yang Y. Xiao W. T. Su Q. (2016). Validity and reliability of the Chinese version of the cognitive flexibility inventory in college students. *Chin. Ment. Health J.* 30 58–63. 10.3969/j.issn.1000-6729.2016.01.012

[B83] Weber A. Cutler A. (2004). Lexical competition in non-native spoken-word recognition. *J. Mem. Lang.* 50 1–25. 10.1016/S0749-596X(03)00105-0

[B84] Weiss D. J. Gerfen C. Mitchel A. D. (2009). Speech segmentation in a simulated bilingual environment: a challenge for statistical learning? *Lang. Learn. Dev.* 5 30–49. 10.1080/15475440802340101 24729760 PMC3981102

[B85] Wilson C. Mihalicek V. (2011). *Language Files: Materials for an Introduction to Language and Linguistics.* Columbus, OH: Ohio State University Press.

[B86] Xie Z. Dong Y. (2017). Contributions of bilingualism and public speaking training to cognitive control differences among young adults. *Biling. Lang. Cogn.* 20 55–68. 10.1017/S1366728915000474

[B87] Yang Y. Li Y. Wang X. Liu N. Jiang K. Zhang S. (2021). Cognitive inhibition mediates the relationship between ESL listening proficiency and English spoken word segmentation in Chinese learners: a functional near-infrared spectroscopy (fNIRS) study. *J. Neurolinguistics* 59:100987. 10.1016/j.jneuroling.2021.100987

[B88] Ye Z. Zhou X. (2009). Executive control in language processing. *Neurosci. Biobehav. Rev.* 33 1168–1177. 10.1016/j.neubiorev.2009.03.003 19747595

